# Predictive Models for the Medical Diagnosis of Dengue: A Case Study in Paraguay

**DOI:** 10.1155/2019/7307803

**Published:** 2019-07-29

**Authors:** Jorge D. Mello-Román, Julio C. Mello-Román, Santiago Gómez-Guerrero, Miguel García-Torres

**Affiliations:** ^1^Universidad Nacional de Concepción, Concepción 8700, Paraguay; ^2^Universidad Nacional de Asunción, San Lorenzo 2111, Paraguay; ^3^Universidad Pablo de Olavide, Sevilla 41013, Spain

## Abstract

Early diagnosis of dengue continues to be a concern for public health in countries with a high incidence of this disease. In this work, we compared two machine learning techniques: artificial neural networks (ANN) and support vector machines (SVM) as assistance tools for medical diagnosis. The performance of classification models was evaluated in a real dataset of patients with a previous diagnosis of dengue extracted from the public health system of Paraguay during the period 2012–2016. The ANN multilayer perceptron achieved better results with an average of 96% accuracy, 96% sensitivity, and 97% specificity, with low variation in thirty different partitions of the dataset. In comparison, SVM polynomial obtained results above 90% for accuracy, sensitivity, and specificity.

## 1. Introduction

According to the World Health Organization, the incidence and prevalence of dengue have been increasing in endemic areas of the tropical and subtropical regions. Based on mathematical estimates of the model, approximately 50 million infections occur each year [1, 2], and historically, the Southern Cone (Argentina, Brazil, Chile, Paraguay, and Uruguay) is the subregion that contributes between 50–60% of the dengue cases in the Americans [3, 4].

Early diagnosis of dengue disease is a recurrent need in the world public health system, and machine learning techniques can help doctors diagnose and predict diseases at an early stage, helping not only in improving the accuracy performance of classification but also in saving diagnostics time, cost, and the pain accompanying pathology tests [5, 6]. Raval et al. [7] point out that the machine learning techniques have been used in medical diagnosis allowing the disease to be analysed based on clinical and laboratory symptoms, providing an accurate result. They also point out that artificial neural network (ANN) is one of the main techniques used to solve medical diagnostic problems, and also the support vector machines (SVM) provide accurate results when evaluating a single disease.

ANN has been applied in several studies of dengue forecast models. In [8], Ibrahim et al. use clinical and epidemiological data from Malaysia. In another work, Cetiner et al. [9] apply ANN to weather data from the Singaporean National Environment Agency (SNEA). A weather dataset was also used by Rachata et al. [10], and feature selection algorithms were applied to predict the incidence of dengue in Thailand.

SVM is another technique quite popular to address this problem. Wu et al. [11] apply SVM to Singapore weather dataset to predict dengue fever. Gomes et al. [12] use gene expression data and apply a radial basis function (RBF) kernel on a small sample from Brazil. Support vector regression (SVR) is also applied by Guo et al. [13]. In this case, they compare several machine learning approaches to data collected from the Guangdon region (China). In a more recent work, Carvajal et al. [14] study the incidence of dengue in Philippines using meteorological factors. They compare several machine learning techniques such as general additive modelling, seasonal autoregressive integrated moving average with exogenous variables, random forest, and gradient boosting.

In this work, the objective is to compare the performance of ANN and SVM classification models to predict the presence of dengue disease, in terms of accuracy, specificity, and sensitivity [15, 16]. The models were trained in a real dataset of patients admitted because of fever in health centres of northern Paraguay, prediagnosed with dengue, and subsequently confirmed or discarded by means of laboratory criteria during the period 2012–2016.

## 2. Classification Models

### 2.1. Artificial Neural Network (ANN)

Artificial neural networks are, as their name indicates, computational networks which attempt to simulate, in a gross manner, the decision process in networks of nerve cell (neurons) of the biological (human or animal) central nervous system [17, 18].

**Figure 1:**
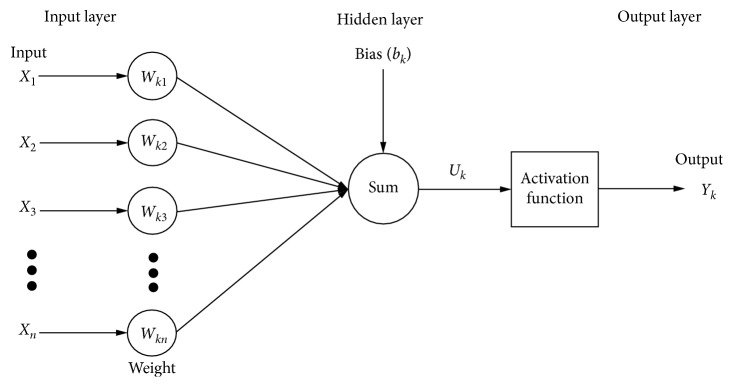
Example of a feedforward architecture with one hidden layer.

The ANN structure consists of three main layers as shown in Figure 1. First, an input layer, which connects the input signal (*X*_*j*_) to the neuron via a set of weights (*W*_*kj*_). Next, a hidden layer, which summarizes the bias values (*b*_*k*_) and the input signals, is weighted by the respective weight values of the neuron. Finally, an output layer is used for limiting the amplitude of the output of the neuron using the activation transfer function. In addition, a bias is added to the neuron to increase or decrease the net output of the neuron [19].

The mathematical structure of a neuron *k* in ANN is represented as follows [20, 21]:

(1) $$\begin{array}{c} U_{k}=\overset{n}{\underset{j=1}{\sum }}\left(W_{kj}X_{j}\right),\\ Y_{k}=f\left(U_{k}+b_{k}\right), \end{array}$$

where *X*_*j*_ are the input signals, *W*_*kj*_ are the weights for neuron *k*, *b*_*k*_ is the bias value, *U*_*k*_ is the linear combiner, *f*(·)  is the activation transfer function, and *Y*_*k*_ is the output signal of the neuron. Two neural networks widely used as supervised training methods are the multilayer perceptron (MLP) and radial basis function (RBF) networks [22, 23].

MLP is a network formed by an input layer, at least one hidden layer and an output layer, trained with the use of the backpropagation learning algorithm, which consists of using the error generated by the network and propagating it backwards. RBF is a neural network that uses radial basis functions as activation functions. There are different types of radial basis functions, but the most widely used type is the Gaussian function. The architecture used by the RBF is very similar to that of the multilayer perceptron, with the characteristic that the RBF always uses three layers: an entry layer, a hidden layer, and an exit layer, while MLPs may have more [24, 25]. A description in some more detail of the different types of ANN systems can be found in [26].

### 2.2. Support Vector Machine (SVM)

**Figure 2:**
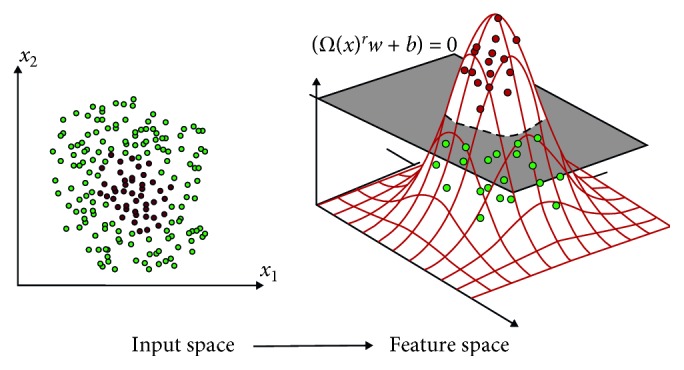
Example of nonlinear SVM [29].

SVM is a classification technique that has given satisfactory results in many practical applications. It also works very well with high dimensional data. SVM looks for a separator hyperplane with the highest margin [27, 28]. An example of nonlinear SVM is shown in Figure 2.

Consider the nonlinear transformation Φ : *R* ^*m*^⟶*ℋ* that allows to project the input vectors from the original coordinate space *x* to a transformed space Φ(*x*) ∈ *ℋ*, provided with scalar product. The scalar product between two given vectors in the transformed space enunciated in terms of a function of similarity in the original space Φ(*u*) · Φ(*v*)=*K*(*u*, *v*) is known as the kernel function [30, 31].

Some well-known kernel functions are as follows:

- Linear *K*(*u*, *v*)=*u* · *v*
- Polynomial *K*(*u*, *v*)=(*u* · *v*+*b*)^*p*^
- Gaussian *K*(*u*, *v*)=exp(−*σ*‖*u* − *v*‖^2^)

Consider the problem of binary classification consisting of *N* examples of training. Each example is indicated by a tuple (*X*_*i*_, *y*_*i*_), where *X*  corresponds to the set of attributes for example *i* and the class denomination is indicated by *y*_*i*_ ∈ {1, −1}. The learning task with SVM can be formalized as the following constrained optimization problem [32, 33]:

(2) $$\begin{array}{c} \text{maximise }L=\overset{N}{\underset{i}{\sum }}\lambda_{i}+\frac{1}{2}\underset{i,j}{\sum }\lambda_{i}\lambda_{j}y_{i}y_{j}K\left(X_{i},X_{j}\right), \end{array}$$

such that ∑_*i*_^*N*^ *λ*_*i*_ *y*_*i*_=0 and *λ*_*i*_ ≥ 0 for all *i*.

A test case *Z* can be classified using the equation *f*(*z*)=sign(∑_*i*_^*n*^ *λ*_*i*_ *y*_*i*_ *K*(*X*_*i*_, *Z*)+*b*), where *λ*_*i*_ is a Lagrange multiplier, *b* is a parameter, and *K* is a kernel function.

### 2.3. Evaluation of Classification Models

In the medical sciences, there are many measures used to calculate indices related to diagnostic accuracy: sensitivity, specificity, and others [34]. In this paper, to evaluate the performance of classification models ANN and SVM, the accuracy, sensitivity, and specificity of the confusion matrices obtained for the test datasets will be calculated [15] and the variation thereof in several test sets obtained from randomised partitions of the dataset.

The accuracy of a test is its ability to differentiate the patient and healthy cases correctly, the sensitivity is its ability to determine the patient cases correctly, and the specificity is its ability to determine the healthy cases correctly. Mathematically, this can be stated as [35]

(3) $$\begin{array}{c} \text{accuracy}=\frac{\text{TP}+\text{TN}}{\text{TP}+\text{TN}+\text{FP}+\text{FN}},\\ \text{sensitivity}=\frac{\text{TP}}{\text{TP}+\text{FN}},\\ \text{specificity}=\frac{\text{ TN}}{\text{TN}+\text{FP}}\text{,} \end{array}$$

where TP is the number of cases correctly identified as patient, FP is the number of cases incorrectly identified as patient, TN is the number of cases correctly identified as healthy, and FN is the number of cases incorrectly identified as healthy.

## 3. Dataset

**Figure 3:**
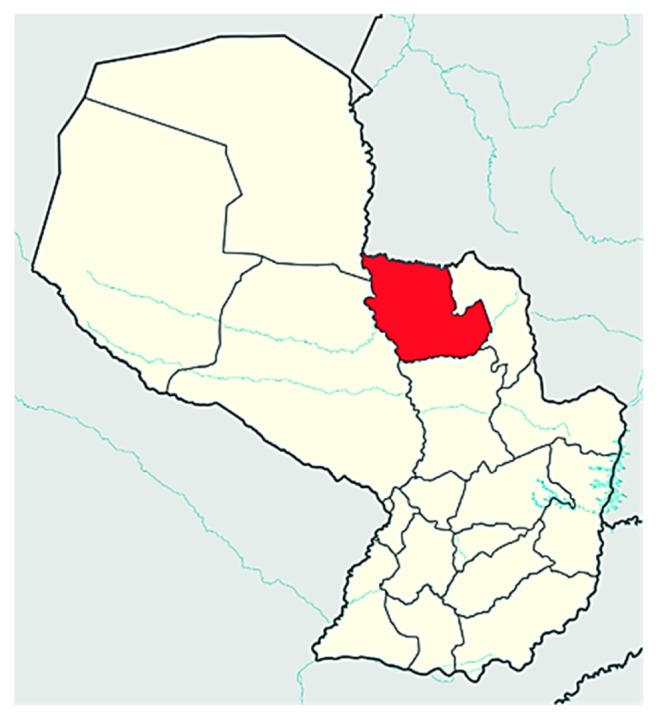
Department of Concepción, Paraguay.

The original dataset is composed of cases registered by the public health system of Paraguay, such as patients admitted for fever and initially diagnosed with dengue, in various health centres of the Department of Concepción, between the years 2012 to 2016. Concepción is located to the northwest of the eastern region of the country, bordering Brazil to the north, as shown in Figure 3.

There were 4332 cases registered, of which 53% corresponded to women and 47% to men. The age of the patients is normally distributed, with the age group between 20 and 39 being the most frequent, 83% of the patients stated that they reside in urban areas, and 55% said that similar cases were occurring simultaneously in their surroundings. Of all the cases, 82% were treated as outpatients.

**Figure 4:**
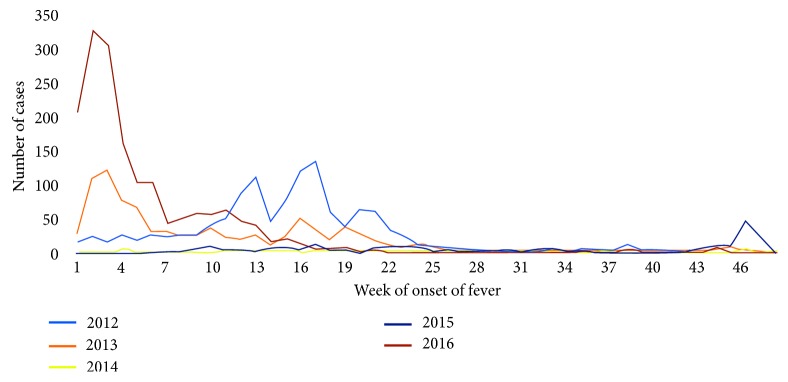
Number of patients diagnosed with dengue by week of onset of fever.

The cases occurred mainly between the months of December and May over the five years.  Figure 4 shows the evolution of the number of patients diagnosed with dengue by week of onset of fever. The datasets related to patients registered in health centres generally have many difficulties throughout the world, characterised by being incomplete, incorrect, scarce, and inaccurate [36, 37]. This dataset is not the exception and leads to the need for an exhaustive preprocessing in order to determine which are the cases and variables that provide useful information for processing.

### 3.1. Dataset Preprocessing

The original dataset was constituted by dengue cases evaluated by one of the laboratory criteria for diagnosis (dengue IgM serological tests or virological tests such as viral isolation or RT-PCR) or by the criterion of the epidemiological link. The epidemiological link is to confirm the probable cases of dengue from laboratory-confirmed cases using the association of person, time, and space [38–40]. Only cases confirmed or discarded with laboratory criteria were included in the study.

In order to ensure minimal loss of information, we proceeded to treat missing values in three stages. First, variables with more than 20% of missing values were excluded. According to [41, 42], it is not recommended to impute data in situations in which the omission in one or more variables reaches percentages higher than an established threshold, because it puts statistical reliability at risk. As a second stage, cases with a nonresponse rate higher than 80% were eliminated [43, 44].

For the selection of the appropriate imputation of missing values, there are no specific rules. It depends on the type of the dataset, file size, nonresponse type, pattern of loss of response, of the research objectives, specific characteristics of the population, general characteristics of the organization of the study, or available software [45]. As the third stage and given the characteristics of the data input process in the dataset and its epidemiological nature, it was considered most appropriate to impute the mean of the valid adjacent data to the missing values.

After treatment of missing values, the final dataset is comprised of 668 cases and the variables included are described in Table 1.

## 4. Implementation and Results

The tests were carried out with the support of the IBM SPSS Modeler software [46]. Firstly, the classification models have been configured in such a way that random partitions of the dataset are carried out by 90% for the training dataset and 10% for the test dataset. For the ANN classifier, the objective has been to increase the accuracy of the model, and the automatic calculation of the number of hidden layers has been arranged for two models of the neuronal network: the multilayer perceptron (MLP) and radial basis function (RBF).

For the SVM classifier, the performance of three kernel functions has been evaluated: linear, Gaussian, and polynomial. Previously, several tests have been carried out to determine the best parameters of the kernel functions and the optimal penalty parameters, based on the accuracy of the classification in the test datasets. The results are described in Table 2.

To evaluate the performance of the ANN and SVM classifiers as predictive models, thirty (30) random partitions of the dataset were performed in the percentages indicated for training and test datasets. The performance of the ANN and SVM classifiers was evaluated exclusively on the test datasets; Table 3 shows the sample mean and the coefficient of variation (the ratio between the sample standard deviation and the sample mean) of the accuracy, sensitivity, and specificity.

It is important to highlight that a 96% sensitivity of the ANN-MLP classifier represents a 4% probability of committing a false-negative type error, which in the medical case is equivalent to diagnosing sick individuals as healthy.

Thirty confusion matrices were obtained for each classifier evaluated, one matrix for each partition of the dataset. The confusion matrices of ANN-MPL and SVM polynomial classifiers, with higher accuracy, are described in Tables 4 and 5.

## 5. Conclusions

We compared the results of two classifiers, ANN and SVM, frequently used in medical sciences as machine learning techniques to assist in medical diagnosis. ANN-MLP and SVM polynomials have shown that they can perform as classifiers in the diagnosis of dengue disease with high averages of accuracy, sensitivity, and specificity, within the spatial and temporal context determined by the dataset.

An important methodological step of this paper is the preprocessing of the dataset considering the particularity of its origin in the public health system and the problems that this entails in the loading of the data. The dataset is validated for the construction of machine learning classifiers.

The results obtained by both classifiers are encouraging for its use in experimental stage and lead to propose more exhaustive investigations with controlled data capturing systems. Additionally, the predictive models generated can be integrated into computer systems to assist in the diagnosis of dengue disease in Paraguay.

## Figures and Tables

**Table 1:** Variables included.

Variables	Description	Measure	Values
Week	Week declared by patient when fever started	Scale	[1,48] ∈ *ℕ*
Year	Registration year	Scale	[1,5] ∈ *ℕ*
Age	Age of the patient at the time of registration	Scale	[0,99] ∈ *ℕ*
Sex	Patient's sex	Nominal	1 = male, 0 = female
District	Geographic area of the Department of Concepción where the patient resides	Nominal	[1,9] ∈ *ℕ*
Hospitalised	Indicates whether the patient was hospitalised or treated as an outpatient	Nominal	1 = yes, 0 = no
Headache	Presence of the symptom	Nominal	1 = yes, 0 = no
Myalgia	Presence of the symptom	Nominal	1 = yes, 0 = no
Arthralgia	Presence of the symptom	Nominal	1 = yes, 0 = no
Retro-ocular pain	Presence of the symptom	Nominal	1 = yes, 0 = no
Pruritus	Presence of the symptom	Nominal	1 = yes, 0 = no
Sickness	Presence of the symptom	Nominal	1 = yes, 0 = no
Cough and dyspnea	Presence of the symptom	Nominal	1 = yes, 0 = no
Oligoanuria	Presence of the symptom	Nominal	1 = yes, 0 = no
Epistaxis	Presence of the symptom	Nominal	1 = yes, 0 = no
Gingivorrhagia	Presence of the symptom	Nominal	1 = yes, 0 = no
Hemoptysis	Presence of the symptom	Nominal	1 = yes, 0 = no
Melena	Presence of the symptom	Nominal	1 = yes, 0 = no
Black vomiting	Presence of the symptom	Nominal	1 = yes, 0 = no
Exanthema	Presence of the symptom	Nominal	1 = yes, 0 = no
Shock	Presence of the symptom	Nominal	1 = yes, 0 = no
Conjunctival injection	Presence of the symptom	Nominal	1 = yes, 0 = no
Bipalpebral edema	Presence of the symptom	Nominal	1 = yes, 0 = no
Tachycardia	Presence of the symptom	Nominal	1 = yes, 0 = no
Hepatomegaly	Presence of the symptom	Nominal	1 = yes, 0 = no
Splenomegaly	Presence of the symptom	Nominal	1 = yes, 0 = no
Sensory alteration	Presence of the symptom	Nominal	1 = yes, 0 = no
Stiff neck	Presence of the symptom	Nominal	1 = yes, 0 = no
Petechia	Presence of the symptom	Nominal	1 = yes, 0 = no
Purpura	Presence of the symptom	Nominal	1 = yes, 0 = no
Jaundice	Presence of the symptom	Nominal	1 = yes, 0 = no
Other symptoms	Presence of other symptoms	Nominal	1 = yes, 0 = no
Travel	Patient's statement if he/she travelled the last 15 days	Nominal	1 = yes, 0 = no
Camping	Patient's statement if he/she was camping the last 15 days	Nominal	1 = yes, 0 = no
Similar health condition	Patient's statement if he/she had a similar health condition before	Nominal	1 = yes, 0 = no
Live alone	Patient's statement if he/she lives alone	Nominal	1 = yes, 0 = no
Poverty	Doctor's impression of whether the patient is in poverty	Nominal	1 = yes, 0 = no
Final classification	Laboratory diagnosis for confirming dengue virus infection	Nominal	*C* = confirmed, *D* = discarded

**Table 2:** Selection of best parameters of the SVM classifier.

Kernel	Penalty	Kernel parameters
Linear	*C* = 10	—
Gaussian	*C* = 11	*σ*=0.01
Polynomial	*C* = 10	*p*=3; *b*=0

**Table 3:** The performance of the ANN and SVM classifiers on 30 test datasets.

Classifiers	Accuracy (%)		Sensitivity (%)		Specificity (%)	
	Mean	Variation	Mean	Variation	Mean	Variation
ANN-MLP	96	2	96	4	97	3
ANN-RBF	55	11	58	10	52	24
SVM linear	64	8	56	18	71	10
SVM Gaussian	86	9	84	11	89	10
SVM polynomial	92	14	93	12	92	16

The ANN-MLP classifier achieved better results with an average of 96% accuracy, 96% sensitivity, and 97% specificity, with low variation in thirty different partitions of the dataset. Also, the SVM polynomial classifier obtained results above 90% for the three indicators with acceptable variations.

**Table 4:** ANN-MLP confusion matrix.

Laboratory diagnosis of dengue virus infection	Predicted	
	Confirmed	Discarded
Confirmed	36	0
Discarded	1	43

**Table 5:** SVM polynomial confusion matrix.

Laboratory diagnosis of dengue virus infection	Predicted	
	Confirmed	Discarded
Confirmed	45	0
Discarded	1	33

## Data Availability

The data used to support the findings of this study are available from the corresponding author upon request.
